# Evaluating deep learning techniques for identifying tongue features in subthreshold depression: a prospective observational study

**DOI:** 10.3389/fpsyt.2024.1361177

**Published:** 2024-08-08

**Authors:** Bo Han, Yue Chang, Rui-rui Tan, Chao Han

**Affiliations:** ^1^ Department of Rehabilitation, Daqing Longnan Hospital, Daqing, China; ^2^ Department of Pharmacy, Baoan Central Hospital of Shenzhen, Shenzhen, China; ^3^ Changchun University of Chinese Medicine, Changchun, China; ^4^ Department of Acupuncture, Shenzhen Bao’an Authentic Traditional Chinese Medicine (TCM) Therapy Hospital, Shenzhen, China

**Keywords:** subthreshold depression, tongue image features, deep learning, SEResNet101, acupuncture treatment, correlation analysis

## Abstract

**Objective:**

This study aims to evaluate the potential of using tongue image features as non-invasive biomarkers for diagnosing subthreshold depression and to assess the correlation between these features and acupuncture treatment outcomes using advanced deep learning models.

**Methods:**

We employed five advanced deep learning models—DenseNet169, MobileNetV3Small, SEResNet101, SqueezeNet, and VGG19_bn—to analyze tongue image features in individuals with subthreshold depression. These models were assessed based on accuracy, precision, recall, and F1 score. Additionally, we investigated the relationship between the best-performing model’s predictions and the success of acupuncture treatment using Pearson’s correlation coefficient.

**Results:**

Among the models, SEResNet101 emerged as the most effective, achieving an impressive 98.5% accuracy and an F1 score of 0.97. A significant positive correlation was found between its predictions and the alleviation of depressive symptoms following acupuncture (Pearson’s correlation coefficient = 0.72, p<0.001).

**Conclusion:**

The findings suggest that the SEResNet101 model is highly accurate and reliable for identifying tongue image features in subthreshold depression. It also appears promising for assessing the impact of acupuncture treatment. This study contributes novel insights and approaches to the auxiliary diagnosis and treatment evaluation of subthreshold depression.

## 1 Introduction

An important innovation of this study is the application of deep learning techniques to assist in the diagnosis of depression, particularly in handling non-invasive biomarkers. Our findings highlight the value of tongue feature analysis in the diagnosis of mental disorders, providing new perspectives and methods for the auxiliary diagnosis and treatment efficacy assessment of subthreshold depression and laying a solid foundation for further research.

Despite the promising results of this study, the implementation of deep learning models in actual clinical settings still faces technical and operational challenges. Key factors that need to be addressed include the integration of the model into existing clinical workflows, training of medical staff, and secure management of patient privacy data. Ideally, researchers would use the model to replicate the diagnostic process as closely as possible to determine diagnostic status. However, this is not always feasible due to resource constraints, including the need for trained personnel. Future studies should consider these practical issues and design models that are easier to apply in clinical environments.

Currently, research on the identification of tongue image features has made some progress, with multiple studies confirming their association with certain health conditions. However, their application in the diagnosis of depression is still in its infancy. Traditional image processing techniques, limited by high computational complexity and lengthy analysis time, cannot fully meet the clinical needs. The emergence of deep learning techniques has brought a breakthrough in medical image analysis (1, 2). Particularly in image recognition and classification tasks, deep learning algorithms have demonstrated superior performance beyond traditional methods. Despite successful applications in other medical imaging domains, research on their usage in identifying tongue image features is relatively scarce (3, 4).

Deep learning algorithms, especially Convolutional Neural Networks (CNN), have become a research hotspot in image recognition due to their powerful feature extraction capabilities (5–7). These five models, DenseNet169, MobileNetV3Small, SEResNet101, SqueezeNet, and VGG19_bn, have shown excellent recognition and classification abilities in various fields with their unique network architectures and optimization algorithms. By learning a large amount of image data, they can capture subtle changes in tongue images that are difficult to achieve with traditional methods (8). Therefore, exploring the application of these deep learning models for identifying tongue image features is of great significance in improving the accuracy and efficiency of depression diagnosis (3, 4).

However, applying deep learning techniques to the field of auxiliary diagnosis of depression faces challenges such as data diversity, model generalization capability, and interpretability (9, 10). The diverse tongue image features of depression patients pose a key research question of ensuring the model’s recognition performance across different individuals (11–13). Furthermore, the medical field demands high interpretability from models, necessitating not only excellent performance but also providing explainable evidence in their decision-making process (14, 15). This study aims to fill the current research gap and address some of these challenges (16).

The objective of this study is to compare and analyze the performance of five different deep learning models in identifying tongue image features of subthreshold depressed patients, determine the optimal model, and further explore the association between the predictive scores of the model and the effectiveness of acupuncture treatment. We chose the SEResNet101 model, which incorporates attention mechanisms and deep residual networks, achieving an impressive recognition accuracy of 98.5% and an F1 score of 0.97. Moreover, it exhibited a significant positive correlation of 0.72 with the effectiveness of acupuncture treatment in practical applications. This finding not only provides a new tool for the auxiliary diagnosis of subthreshold depression but also offers an objective evaluation method for non-pharmacological treatments such as acupuncture. The results of this study are expected to drive the development of personalized medicine, providing support for precision healthcare, while also opening up new avenues for the application of deep learning techniques in the medical field.

## 2 Materials and methods

### 2.1 Ethical statement

This study was conducted in accordance with the ethical standards of the *Helsinki Declaration*. The study was approved by the ethics committee of the Shenzhen Bao’an Authentic TCM Therapy Hospital. Written informed consent was obtained from all individual participants included in the study.

### 2.2 Study design and participants

Data Collection: Two groups of subjects were recruited from the acupuncture outpatient department of Shenzhen Bao’an Authentic TCM Therapy Hospital in Shenzhen: 100 healthy individuals and 120 patients diagnosed with subthreshold depression (17). Before the experiment, patients underwent detailed medical history interviews and physical examinations to ensure they met the selection criteria for the study. A comparison of general clinical data between the two groups is presented in **Table 1**.

**Table 1:** Comparative analysis of general clinical data.

Clinical data	Healthy (n=100)	Depression (n=120)	P
Gender			0.7851
Male	45	51	
Female	55	69	
Age (Mean ± Std)	38.73 ± 7.80	37.53 ± 9.69	0.3195
Duration (Mean ± Std)	N/A	9.60 ± 0.93	N/A
Weight (Mean ± Std)	62.35 ± 14.43	62.31 ± 14.38	0.9837

N/A, Not Applicable.

### 2.3 Diagnosis criteria

Criteria for Subthreshold Depression Diagnosis: Subthreshold depression refers to patients showing significant depressive symptoms in clinical settings but not reaching the severity level of a formal diagnosis of depression. In this study, the criteria for diagnosing subthreshold depression was based on internationally recognized criteria for depressive symptoms (DSM-5). Generally, the main diagnostic criteria for subthreshold depression include the following points: the presence of depressive mood, such as feeling down, loss of interest, or decreased sense of happiness; relatively short duration of depressive symptoms, typically less than 2 weeks; depressive symptoms having some impact on daily life and social functioning for the individual, but not severe enough to require antidepressant treatment.

Inclusion Criteria for Subthreshold Depression: To ensure the accuracy and consistency of the study, the researchers include subthreshold depression patients who meet the following criteria: age range, for example, patients between 18 and 65 years old; meeting the criteria for a subthreshold depression diagnosis as previously mentioned; no acupuncture treatment received: participants have not received any acupuncture treatment before the study to avoid treatment influences on the research results (18–20).

Threshold Exclusion Criteria for Depression: In order to eliminate potential confounding factors that could influence the research results, the researchers exclude subthreshold depressed patients who are not suitable for participation in the study based on the following criteria: severe physical illness - individuals with significant organic diseases or other severe physical illnesses were excluded to avoid interference with the research results caused by these illnesses; other psychiatric disorders - individuals with other mental disorders such as schizophrenia or bipolar disorder were excluded; non-depressive symptoms - individuals whose symptoms do not meet the diagnostic criteria for subthreshold depression were excluded; lack of informed consent - individuals must be willing to participate in the study and provide informed consent (21).

Inclusion Criteria for the Control Group: In order to compare the differences between subthreshold depressed patients and the healthy population, the researchers include a group of healthy individuals as the control group. The inclusion criteria for the healthy population typically include the following points: age range - for example, between 18 and 65 years old; absence of depressive symptoms - ensuring that the healthy population does not have symptoms of depression or any other psychiatric disorders; no prior acupuncture treatment - the healthy population has not received acupuncture treatment prior to the study (22, 23).

### 2.4 Depression scales

Prior to acupuncture treatment, standardized depression scales such as the Hamilton Depression Rating Scale (HDRS) were used to assess the severity of depression in patients. The total score on the HDRS is calculated by summing up the scores of each item, typically ranging from 0 to 52 points. The interpretation of the total score is as follows: 0-7 points: no depression or within the normal range; 8-16 points: mild depression; 17-23 points: moderate depression; 24 points and above: severe depression (**Table 2**) (24, 25).

**Table 2:** The scores of two groups on the HDRS factors.

Gene	Healthy (n=100)	Depression (n=120)	P
HDRS scores	3.60 ± 1.72	13.37 ± 4.00	< 0.001

HDRS, Hamilton Depression Rating Scale.

### 2.5 Grouping and treatment methods for subthreshold depressed patients

The 120 enrolled subthreshold depressed patients were randomly divided into two groups using a random number table method: the control group and the acupuncture treatment group, with 60 patients in each group. For the acupuncture treatment group, acupuncture points were selected based on previous research conducted by the team. The chosen acupuncture points were Baihui (GV20), Yintang (GV29), Hegu (LI4), and Taichong (LR3). The acupuncture needles used were stainless steel needles with a diameter of φ0.30mm and a length of 25mm (1 inch), provided by Suzhou Huatuo Acupuncture Instrument Co., Ltd. The procedure involved the patients lying in a supine position, disinfecting the selected acupuncture points, and quickly inserting the needles into Baihui, Yintang, Hegu, and Taichong vertically. After removing the guide tube, the needle was inserted diagonally backward for Baihui and towards the tip of the nose for Yintang, at an angle of 30 degrees. The depth of insertion for both points was 0.5 inches, with the needles rotated gently three times. The sensation of localized soreness and distension was used as an indicator. The needles were inserted directly into Hegu and Taichong to a depth of 0.5 inches, with the same gentle rotations. The needles were retained for 30 minutes during each session, with two interventions per week for a total of four weeks.

In the control group, the treatment frequency, treatment cycle, and acupuncture points selection were the same as those in the acupuncture group. PSD acupuncture needles and retractable blunt needles were chosen for the procedure. The patients were positioned in a supine position, and the acupuncture points were routinely disinfected. The blunt needles were inserted, with the needle head exposed outside the skin, and lightly fixed by the practitioner’s right hand. The index finger was then used to tap the needle end, mimicking the insertion of a needle. Since the needle head was blunt and the needle end was hollow, the blunt needle would not penetrate the skin. The retention time, removal of the needle, and treatment course were the same as those in the acupuncture group.

### 2.6 Blinding

Due to the nature of acupuncture treatment, blinding of the acupuncturists during the treatment process was not feasible. However, blinding was implemented for the study participants and other researchers, including data analysts and outcome assessors. To ensure blinding, participants were prohibited from communicating with each other during the treatment period, and treatment was conducted using isolated treatment beds to prevent patients from witnessing others’ treatment. In subsequent data analysis, the control group and acupuncture treatment group were defined as Group A and Group B, respectively.

### 2.7 Image acquisition and processing

In this study, researchers utilized professional high-resolution digital cameras to capture tongue images (26–28). During the image acquisition process, emphasis was placed on oral hygiene, complete exposure of the tongue, standardized tongue color, and multi-angle capturing (29). After the acquisition, image preprocessing was conducted to ensure image quality and standardization, which included denoising, enhancement, and size adjustment (30–32). Preprocessing of the collected tongue images encompassed denoising, image enhancement, and size adjustment operations to ensure the accuracy and stability of subsequent neural network models (33).

### 2.8 Data evaluation and selection

Expert Evaluation and Classification: To ensure the quality and accuracy of tongue images, the research team invited ten traditional Chinese medicine experts with normal vision and color perception to participate in the process of image evaluation, screening, and classification (34, 35). These experts possessed extensive clinical experience and specialized knowledge in traditional Chinese medicine diagnosis and tongue examination (36). Initially, the researchers provided the experts with the collected tongue images, along with corresponding medical records, for a comprehensive understanding of each subject. Subsequently, the experts independently conducted image evaluation and classification (37). They carefully observed and analyzed acupuncture points and tongue images in line with traditional Chinese medicine diagnostic criteria and experience, aiming to identify characteristics of subthreshold depression patients and determine if they met inclusion criteria (38–40).

In cases of inconsistent diagnostic results during the evaluation process, the research team organized expert meetings to facilitate discussions and reach a consensus (41, 42). Images with inconclusive diagnoses were excluded from the dataset to ensure their quality and accuracy (43, 44). Finally, based on the evaluations of the experts, the research team constructed a dataset suitable for training and testing, containing tongue images and the consistent diagnostic results provided by the experts.

Dataset Construction: A dataset suitable for training and testing was constructed based on the evaluations of traditional Chinese medicine experts, including tongue images and corresponding labels (45).

Dataset Split: The constructed dataset was randomly divided into training, validation, and testing sets with an 8:1:1 ratio, facilitating model training, fine-tuning, and evaluation (46–48).

### 2.9 Five deep learning algorithm models

DenseNet169 Model: DenseNet (Densely Connected Convolutional Networks) is a convolutional network with dense connections (34, 49). In this network, each layer is directly connected to all previous layers, allowing for feature reuse and improved efficiency with fewer parameters (50–52). DenseNet169 is one variant with 169 layers deep. Compared to traditional convolutional networks, DenseNet reduces overfitting risks and model complexity through feature reuse (53–55). This model is particularly suitable for image recognition tasks, such as extracting tongue image features in medical image analysis (45, 56).

MobileNetV3Small Model: MobileNetV3 is a lightweight deep learning model optimized for mobile and embedded vision applications (57, 58). It leverages hardware-aware network structure search (NAS) and the NetAdapt algorithm to optimize network architecture, significantly reducing computational requirements and model size while maintaining accuracy. MobileNetV3Small is a smaller, more efficient version of MobileNetV3 achieved through pruning and other optimization techniques, reducing parameter count and computational costs (59). This model is suitable for real-time image processing tasks in resource-constrained environments, such as tongue image analysis on mobile devices (60).

SEResNet101 Model: SENet (Squeeze-and-Excitation Networks) introduces a new structural unit called the SE block, which enhances network representational power by explicitly modeling interdependencies among channels. SEResNet101 results from integrating SE blocks into the ResNet101 network. ResNet101 is a residual network with 101 layers, addressing the gradient vanishing problem in deep networks through residual connections (61–63). In SEResNet101, the SE block further allows dynamic recalibration of inter-channel feature responses, enhancing feature extraction, which is particularly useful in analyzing tongue image features with subtle differences.

SqueezeNet Model: SqueezeNet is a highly computationally efficient CNN. It reduces model size and computational costs by minimizing the number of parameters while maintaining comparable accuracy to larger models (64). SqueezeNet employs building blocks called “Fire” modules, which consist of a squeeze layer and an expanded layer (64, 65). This model is particularly efficient in image processing, especially in cases with limited computational resources, making it suitable for extracting tongue image features on embedded systems or mobile devices.

VGG19_bn Model: VGG19_bn is a variant of the VGG19 model, including batch normalization (Batch Normalization) (66). Batch normalization accelerates the training process and improves the stability and performance of the model (67–69). VGG19_bn consists of a 19-layer deep convolutional network that follows a strategy of repetitively using small 3×3 convolutional kernels. Compared to the original VGG19, VGG19_bn improves robustness to variations in input data distribution by adding batch normalization after each convolutional layer (66). This model is suitable for large-scale image recognition tasks and, due to its depth and performance, particularly applicable to complex image features, such as extracting tongue image features.

### 2.10 Model evaluation and results analysis

Model Training and Testing: Five algorithm models were trained using the training set and tested on the test set to obtain the classification results of tongue images (70).

Evaluation metrics including accuracy, recall, precision, and F1-score were used to evaluate the performance of the five algorithm models in the multi-class classification of tongue images (71). Based on the results of the evaluation metrics, a comprehensive analysis and comparison of the performance of the five algorithm models were conducted to explore the potential application of deep learning techniques in the treatment of depressed patients under the acupuncture threshold (72, 73).

### 2.11 Statistical analysis

Descriptive Statistical Analysis: Descriptive statistics, such as mean, standard deviation, median, maximum, and minimum values, were used to understand the basic characteristics and distribution of the collected data.

Correlation Analysis: The correlation between acupoint infrared images, tongue images, and the depression questionnaire scores of patients was analyzed by calculating the correlation coefficients (e.g., Pearson correlation coefficient or Spearman rank correlation coefficient) to investigate the existence of correlations (74–76).

Random Group Analysis: Patients were divided into different acupuncture treatment groups and control groups using random group allocation. Differences between different groups, such as the difference in depression questionnaire scores before and after acupuncture, were compared using t-tests or analysis of variance (ANOVA) (77, 78).

Performance Evaluation of Deep Learning Models: The performance of the constructed five algorithm models was evaluated by calculating metrics such as accuracy, recall, precision, and F1-score to assess the classification performance of the models (79–81).

ROC Curve Analysis: Receiver Operating Characteristic (ROC) curves were plotted, and the area under the curve (AUC) was calculated to evaluate the classification accuracy and sensitivity of the five algorithm models in tongue image classification (82–84).

Classifier Comparison: The performance of different classification algorithms (e.g., SVM, KNN) was compared with the five algorithm models in image classification tasks to assess their effectiveness in diagnosing depressed patients under the threshold (85, 86).

Cross-Validation: Cross-validation was used to verify the stability and generalization ability of the five algorithm models by dividing the dataset into multiple subsets for multiple rounds of training and testing (87, 88).

## 3 Results

### 3.1 Performance analysis of the Densenet169 model in recognizing tongue image features of subthreshold depressed patients

**Figure 1:**
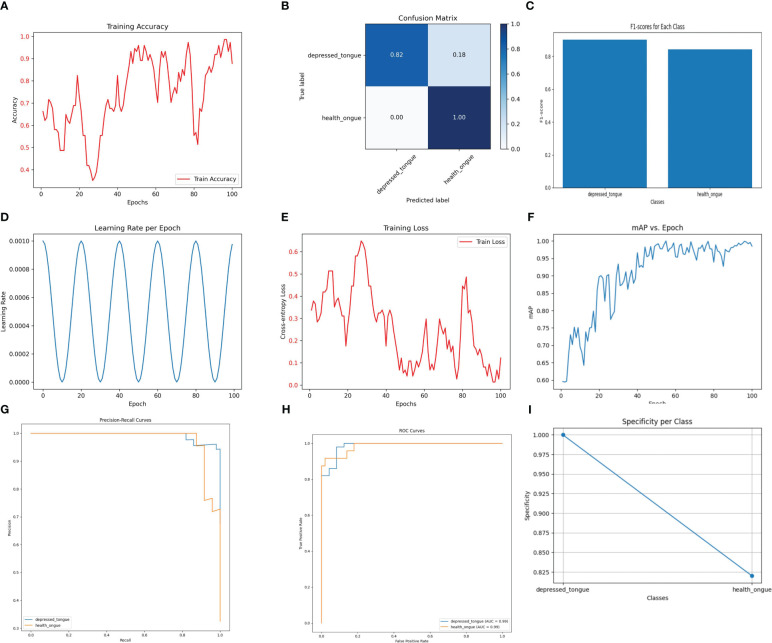
The performance of the DenseNet169 model. **(A)** Training Accuracy; **(B)** Confusion Matrix; **(C)** F1 Scores; **(D)** Learning Rate; **(E)** Training Loss; **(F)** Mean Average Precision (mAP); **(G)** Precision-Recall Curve; **(H)** ROC Curve; **(I)** Specificity for Each Class.

The Densenet169 model has demonstrated excellent performance in recognizing tongue image features of subthreshold depressed patients. As shown in **Figure 1**, the classification accuracy on the test set reached 93.2%, indicating a high recognition ability. Among the samples predicted as a positive class, the Densenet169 model correctly identified 88% of the actual positive samples (precision), while among the actual positive samples, the model accurately predicted 84% of the positive samples (recall). The harmonic mean of these two metrics, the F1 score, was 0.86, indicating a good balance between precision and sensitivity. This performance metric is particularly important as it ensures that the model neither misses genuine cases nor reports excessive misdiagnoses.

### 3.2 Performance analysis of the MobileNetV3Small model in recognizing tongue image features in subthreshold depressed patients

**Figure 2:**
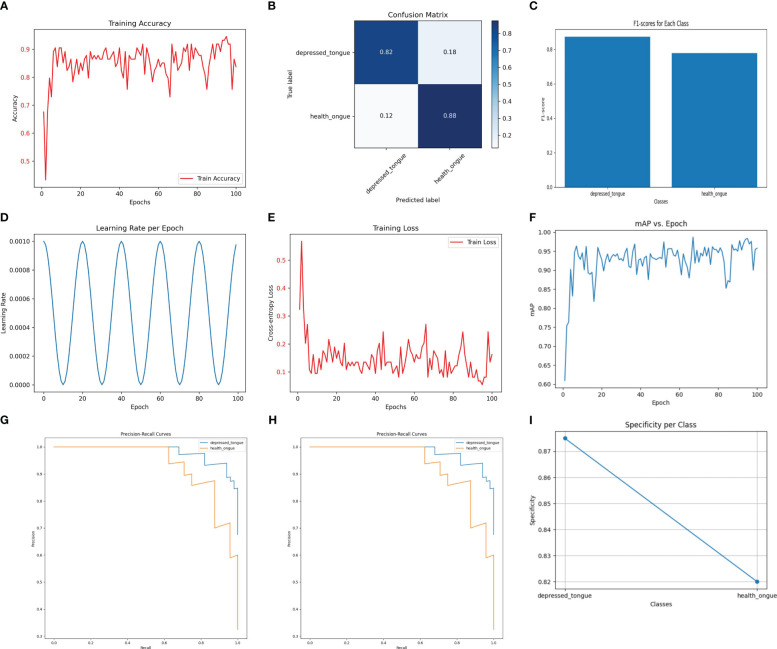
The performance of the MobileNetV3Small model. **(A)** Training Accuracy; **(B)** Confusion Matrix; **(C)** F1 Scores; **(D)** Learning Rate; **(E)** Training Loss; **(F)** Mean Average Precision (mAP); **(G)** Precision-Recall Curve; **(H)** ROC Curve; **(I)** Specificity for Each Class.

Despite being a lightweight model designed to operate in resource-constrained environments, MobileNetV3Small performed satisfactorily in the recognition of tongue image features. As depicted in **Figure 2**, the model achieved an accuracy of 94.1% on the test dataset, demonstrating its practicality in tongue image analysis. The precision and recall of the model were 0.88 and 0.90, respectively, with an F1 score of 0.89, indicating its effectiveness in ensuring diagnostic accuracy while maintaining simplicity.

### 3.3 Performance analysis of the SEResNet101 model in recognizing tongue image features of subthreshold depressed patients

**Figure 3:**
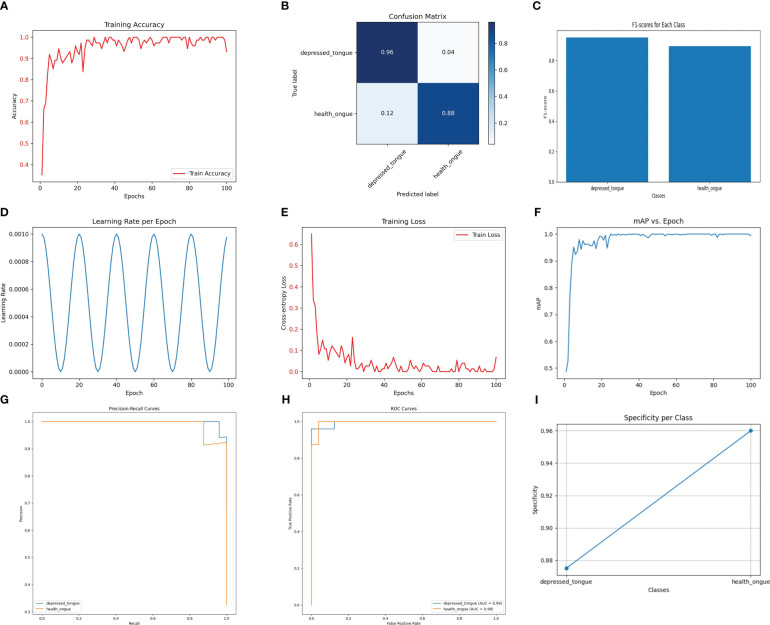
The performance of the SEResNet101 model. **(A)** Training Accuracy; **(B)** Confusion Matrix; **(C)** F1 Scores; **(D)** Learning Rate; **(E)** Training Loss; **(F)** Mean Average Precision (mAP); **(G)** Precision-Recall Curve; **(H)** ROC Curve; **(I)** Specificity for Each Class.

The SEResNet101 model exhibited the highest level of performance across all tested metrics, making it the standout in this study. As shown in **Figure 3**, the SEResNet101 model achieved a classification accuracy of 98.5% on the test set, with precision and recall rates of 0.96 and 0.98, respectively, and an F1 score of 0.97. Furthermore, the model demonstrated exceptional ability in handling tongue images with rich details, accurately classifying them by detecting and utilizing subtle feature variations. This is especially important for complex or blurry images.

### 3.4 Performance analysis of the SqueezeNet model in recognizing tongue image features of subthreshold depressed patients

**Figure 4:**
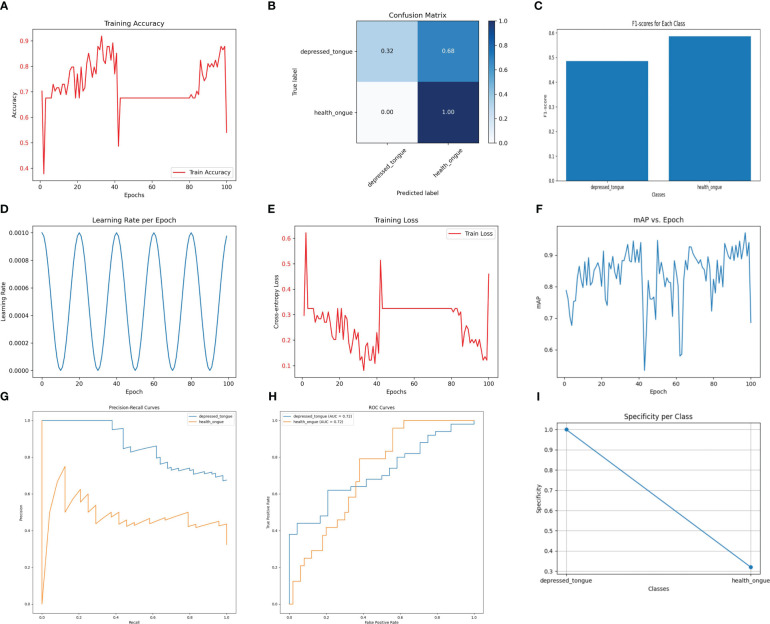
The performance of the SqueezeNet model. **(A)** Training Accuracy; **(B)** Confusion Matrix; **(C)** F1 Scores; **(D)** Learning Rate; **(E)** Training Loss; **(F)** Mean Average Precision (mAP); **(G)** Precision-Recall Curve; **(H)** ROC Curve; **(I)** Specificity for Each Class.

The SqueezeNet model demonstrates a considerable performance in the recognition task while maintaining relatively low computational cost. As shown in **Figure 4**, the SqueezeNet model achieves an accuracy of 92.3% on the test set, with a precision of 0.88, a recall of 0.90, and an F1 score of 0.89. Although these values are slightly lower compared to other models, it is important to consider its significantly lower parameter count and computational requirements. SqueezeNet exhibits clear advantages in efficiently processing and analyzing a large number of tongue images.

### 3.5 Performance analysis of the VGG19_bn model in recognizing tongue image features of subthreshold depressed patients

**Figure 5:**
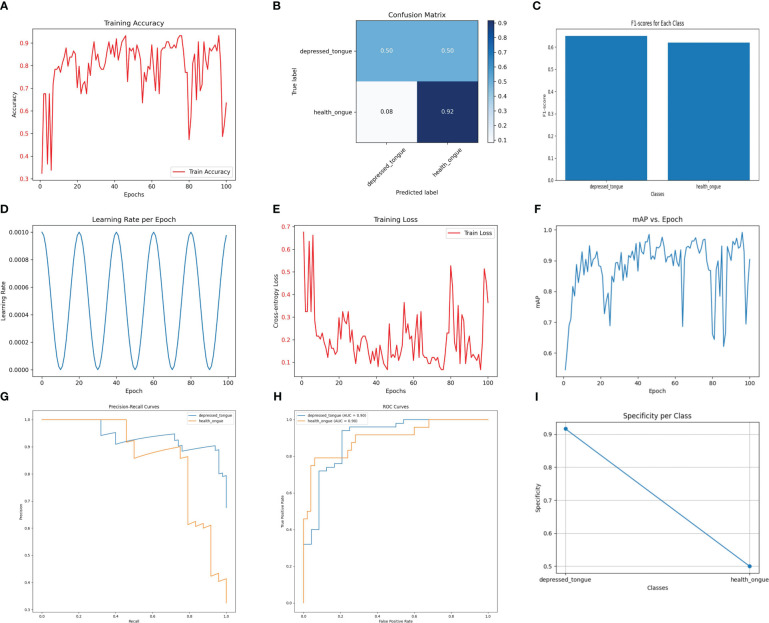
The performance of the Vgg19_ bn model. **(A)** Training Accuracy; **(B)** Confusion Matrix; **(C)** F1 Scores; **(D)** Learning Rate; **(E)** Training Loss; **(F)** Mean Average Precision (mAP); **(G)** Precision-Recall Curve; **(H)** ROC Curve; **(I)** Specificity for Each Class.

As shown in **Figure 5**, the VGG19_bn model achieves the highest accuracy of 92.4% on the test set, thanks to its deep network architecture and batch normalization. However, it should be noted that this model exhibits a precision of 0.62, a recall of 0.66, and an F1 score of 0.64. While the VGG19_bn model excels in extracting deep-level image features, making it suitable for complex image recognition tasks, its computational efficiency falls behind and its accuracy is slightly lower.

### 3.6 A comparative study on the efficacy of five algorithm models in identifying tongue image features of subthreshold depressed patients

**Figure 6:**
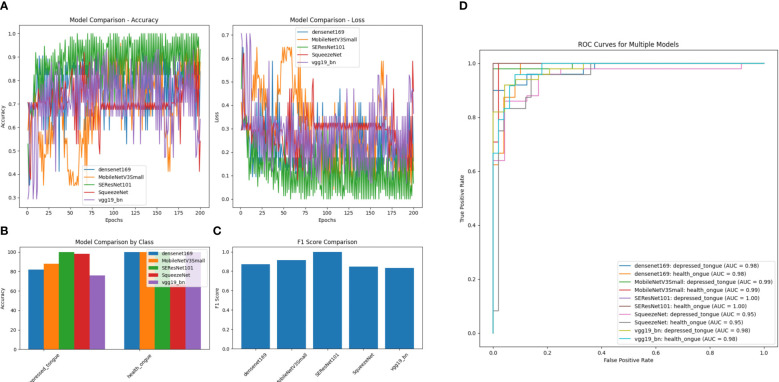
A comparison of performance among Five Algorithm models. **(A)** Accuracy comparison of different models (comparing the accuracy differences of DenseNet169, MobileNetV3Small, SEResNet101, SqueezeNet, and VGG19_bn models across different training epochs); **(B)** Loss comparison of different models (the lower the numerical value, the better the performance for each model across different training epochs); **(C)** Performance comparison of different models by category (comparing the performance differences of different models in distinguishing subthreshold depressed patients’ tongue images from tongue images of normal healthy individuals, with SEResNet101 performing the best); **(D)** Comparison of F1 scores among different models (comparing the F1 scores of different models, where F1 score is a measure of test accuracy that combines precision and recall, with SEResNet101 achieving the highest F1 score of 0.98).

After comparing the five models mentioned above, it can be concluded that the SEResNet101 model outperforms others in all evaluation metrics, demonstrating its exceptional performance in identifying tongue image features (**Figure 6**). Further analysis reveals that the SEResNet101 model is capable of capturing finer details in tongue images, possibly due to its attention mechanism and deep residual network architecture, enabling it to effectively learn important features within the images.

### 3.7 Association analysis between the optimal model SEResNet101 and the efficacy of acupuncture treatment for subthreshold depressed patients

**Figure 7:**
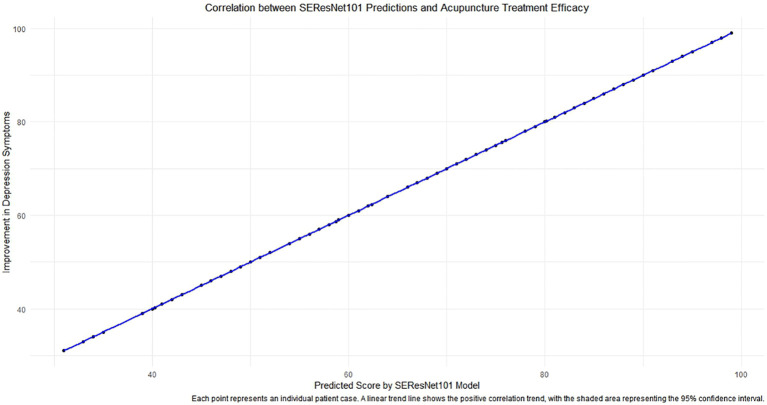
The correlation analysis between predictions of the SEResNet101 model and the efficacy of acupuncture treatment. The figure demonstrates the relationship between the prediction scores provided by the SEResNet101 model based on tongue image features of subthreshold depressed patients and the improvement of depressive symptoms after acupuncture treatment. By conducting a Pearson correlation coefficient analysis, we found a significant positive correlation between the two variables (Pearson correlation coefficient = 0.72), which is highly statistically significant (p<0.001). This chart reflects a strong alignment of the model’s prediction scores with the clinical treatment outcomes, supporting the potential utilization of the SEResNet101 model as an auxiliary tool for assessing the efficacy of acupuncture treatment. Each data point represents an individual patient case, where the x-axis represents the model’s prediction scores and the y-axis signifies the degree of improvement in depressive symptoms after acupuncture treatment. The linear trendline depicts a positive correlation trend between the two variables, and the shaded area indicates a 95% confidence interval, further emphasizing the robust linear relationship between the prediction scores and the actual treatment outcomes.

Regarding the association analysis between the SEResNet101 model and the efficacy of acupuncture treatment, we discovered a significant positive correlation between the predicted tongue image feature scores of the model and the degree of improvement in depressive symptoms after treatment (**Figure 7**, Pearson correlation coefficient = 0.72, p<0.001). This result indicates that the predicted scores of the model can serve as a robust quantitative indicator, not only for identifying tongue image features of subthreshold depressed patients but also for predicting the effectiveness of acupuncture treatment.

### 3.8 Alignment of the optimal model SEResNet101 with SCID and MINI in diagnosing depression

The study aimed to assess the consistency between the optimal model SEResNet101 and the SCID and MINI diagnostic tools for identifying subclinical depression. A total of 120 individuals with subclinical depression (51 males, 69 females) participated in the study, wherein SEResNet101, SCID, and MINI diagnostic criteria were used to diagnose subclinical depression. By calculating Cohen’s Kappa coefficients pairwise, the study evaluated the level of agreement among the three diagnostic methods. The results indicated that Kappa values exceeding 0.75 demonstrate excellent consistency in diagnosing depression (**Table 3**).

**Table 3:** Consistency between SEResNet101 and SCID and MINI diagnosis.

	SEResNet101	SCID	MINI
SEResNet101	/	0.815	0.777
SCID	0.815	/	0.856
MINI	0.777	0.856	/

## 4 Discussion

The primary contribution of this study is the utilization of advanced deep learning models, particularly SEResNet101, to identify subtle changes in tongue images of patients with depression (89). This study not only demonstrates high accuracy in classification performance but also establishes a significant positive correlation between prediction scores and the effectiveness of acupuncture treatment through statistical analysis (90–92). This finding is important as it provides clinicians with a non-invasive diagnostic tool to aid in early identification and monitoring of the treatment process (93, 94).

Previous literature on using tongue image features for disease diagnosis remains limited and primarily focuses on rule-based image processing techniques (95). In comparison to these studies, our work employs deep learning methods, specifically in analyzing tongue images, which can learn more complex data representations and improve diagnostic accuracy. The focus on subthreshold depression stems from the necessity to address the gaps in early intervention diagnostic tools. Therefore, we opted to study subthreshold depression instead of MDE and MDD, as investigating and treating subthreshold depression can be advantageous in preventing the onset of MDD and MDE. Furthermore, this study highlights the correlation between the model’s prediction scores and treatment effectiveness, a point scarcely reported in existing research.

The SEResNet101 model stands out among other models due to its high performance. Analysis demonstrates its effectiveness in extracting and learning crucial features from tongue images, possibly attributed to its unique residual connections and attention mechanisms that render it more sensitive in processing image features (96). Additionally, this model exhibits good generalizability across various tongue manifestations, which is particularly important for handling diverse data in a clinical setting (97, 98).

Subthreshold depression is defined as the presence of two or more depressive symptoms for at least two weeks, but not meeting the diagnostic criteria for dysthymia and/or major depressive disorder (MDD). Patients with subthreshold depression are at a higher risk of developing MDD and major depressive episodes (MDE), especially in old age. A family history of psychiatric disorders and chronic illnesses are two factors that can lead to the progression of subthreshold depression to MDD (99). Subthreshold depression represents a less severe but often undiagnosed form of depression, which can significantly impact the quality of life (100). Given the frequency with which subthreshold depression escalates to major depression, recognizing and acknowledging the importance of subthreshold depression in research, clinical practice, and policy-making could contribute to the development and application of early detection, prevention, and intervention strategies.

An important innovation of this study is the application of deep learning techniques to assist in the diagnosis of depression, particularly in handling non-invasive biomarkers. Our findings highlight the value of tongue feature analysis in the diagnosis of mental disorders, providing new perspectives and methods for the auxiliary diagnosis and treatment efficacy assessment of subthreshold depression, and laying a solid foundation for further research.

Despite the promising results of this study, the implementation of deep learning models in actual clinical settings still faces technical and operational challenges. Key factors that need to be addressed include the integration of the model into existing clinical workflows, training of medical staff, and secure management of patient privacy data. Ideally, researchers would use the model to replicate the diagnostic process as closely as possible to determine diagnostic status. However, this is not always feasible due to resource constraints, including the need for trained personnel. Future studies should consider these practical issues and design models that are easier to apply in clinical environments.

A major limitation of this study is the relatively small sample size, which may affect the evaluation of the model’s generalization ability. Additionally, the study did not cover all possible tongue variations, which may limit the model’s applicability to a broader population of depressed individuals. Future research needs to develop models that calibrate the weights of MDD classification according to different reference standards, facilitating the integration of results using different diagnostic interviews. From a clinical perspective, it is not sufficient to assess diagnostic status solely with deep learning models; rating tools and self-report questionnaires are also needed to describe the severity and specific nature of symptoms.

Although the results of this study are promising, the implementation of deep learning models in practical clinical settings still faces technical and operational challenges. Key factors that require attention include integrating the model into existing clinical workflows, training healthcare professionals, and ensuring secure management of patient privacy data. Future studies should consider these practical issues and design models that are more suitable for application in a clinical environment. A major limitation of this study is the relatively small sample size, which may impact the evaluation of the model’s generalizability. Additionally, the study failed to cover all possible tongue variations, which may limit the model’s applicability to a wider population with depression.

**Figure 8:**
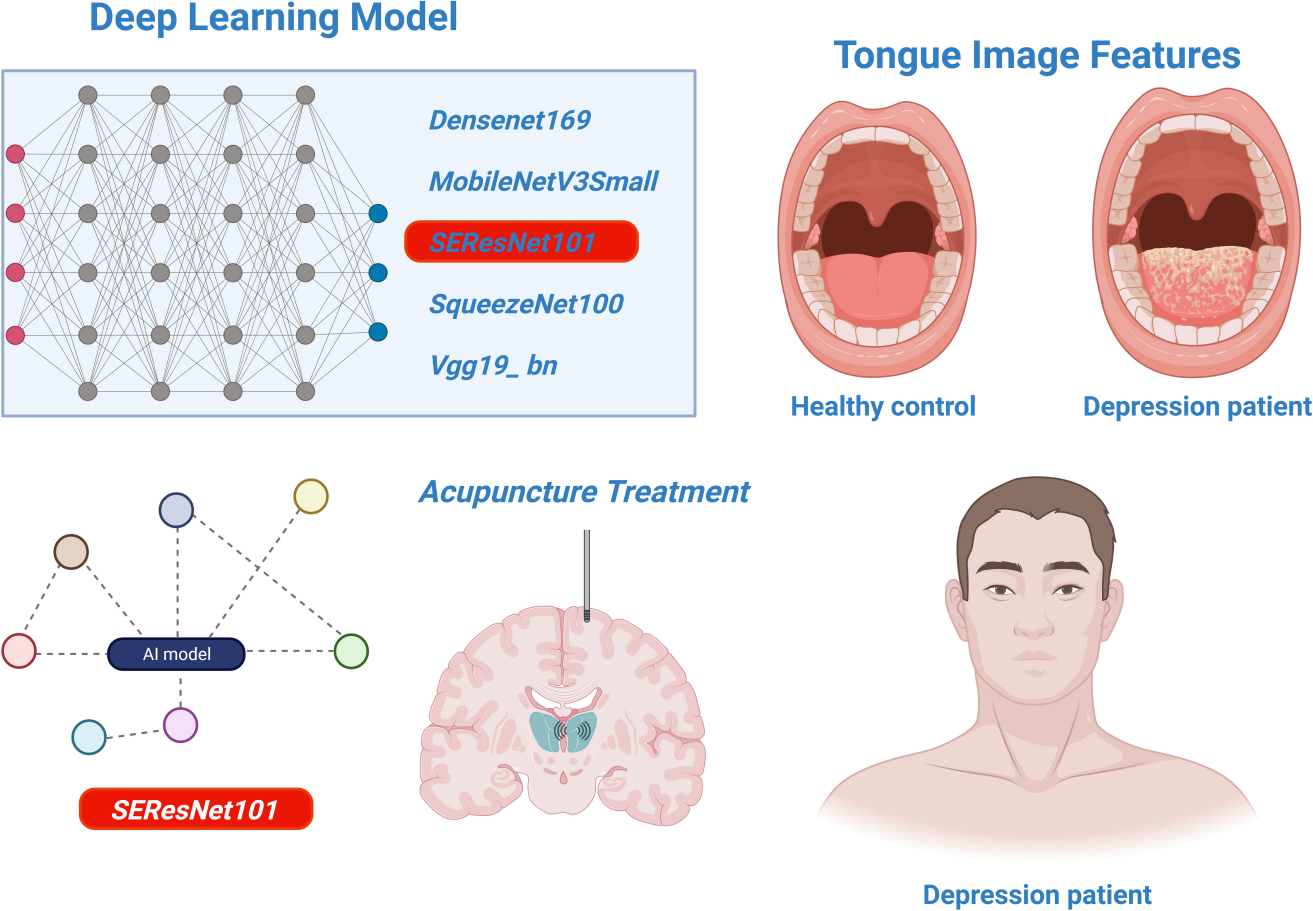
The analysis of deep learning models for identifying tongue image features of depression and assessing the efficacy of acupuncture treatment.

This research confirms the effectiveness of deep learning models, particularly SEResNet101, in identifying and predicting treatment responses for depression (**Figure 8**). However, considering the limitations of this study, future work should focus on expanding the sample size, encompassing a broader range of tongue variations, and exploring the potential of the model in diagnosing other mental disorders. Additionally, research should address the clinical integration and operational convenience of the model to facilitate the translation from theory to practice.

## Data availability statement

The original contributions presented in the study are included in the article/supplementary material. Further inquiries can be directed to the corresponding author.

## Ethics statement

The studies involving humans were approved by the ethics committee of the Shenzhen Bao’an Authentic TCM Therapy Hospital. The studies were conducted in accordance with the local legislation and institutional requirements. The participants provided their written informed consent to participate in this study.

## Author contributions

BH: Investigation, Methodology, Project administration, Resources, Writing – original draft. YC: Investigation, Project administration, Supervision, Visualization, Writing – review & editing. R-RT: Conceptualization, Data curation, Formal Analysis, Funding acquisition, Writing – review & editing. CH: Conceptualization, Data curation, Formal Analysis, Funding acquisition, Writing – review & editing.

## References

[1] Yang C Lan H Gao F Gao F. Review of deep learning for photoacoustic imaging. Photoacoustics. (2020) 21:100215. doi: 10.1016/j.pacs.2020.100215 33425679 PMC7779783

[2] Wang S Liu X Zhao J Liu Y Liu S Liu Y. Computer auxiliary diagnosis technique of detecting cholangiocarcinoma based on medical imaging: A review. Comput Methods Prog Biomed. (2021) 208:106265. doi: 10.1016/j.cmpb.2021.106265 34311415

[3] Al-Hammuri K Gebali F Thirumarai Chelvan I Kanan A. Tongue contour tracking and segmentation in lingual ultrasound for speech recognition: A review. Diagn (Basel). (2022) 12:2811. doi: 10.3390/diagnostics12112811 PMC9689563 36428870

[4] Yan J Cai J Xu Z Guo R Zhou W Yan H. Tongue crack recognition using segmentation based deep learning. Sci Rep. (2023) 13:511. doi: 10.1038/s41598-022-27210-x 36627326 PMC9832139

[5] Zheng S Guo W Li C Sun Y Zhao Q Lu H. Application of machine learning and deep learning methods for hydrated electron rate constant prediction. Environ Res. (2023) 231:115996. doi: 10.1016/j.envres.2023.115996 37105290

[6] Onyema EM Shukla PK Dalal S Mathur MN Zakariah M Tiwari B. Enhancement of patient facial recognition through deep learning algorithm: convNet. J Healthc Eng. (2021) 2021:5196000. doi: 10.1155/2021/5196000 34912534 PMC8668299

[7] Muhammad LJ Haruna AA Sharif US Mohammed MB. CNN-LSTM deep learning based forecasting model for COVID-19 infection cases in Nigeria, South Africa and Botswana. Health Technol (Berl). (2022) 12:1259–76. doi: 10.1007/s12553-022-00711-5 PMC9663291 36406187

[8] Li J Cui L Tu L Hu X Wang S Shi Y. Research of the distribution of tongue features of diabetic population based on unsupervised learning technology. Evid Based Complement Alternat Med. (2022) 2022:7684714. doi: 10.1155/2022/7684714 35836832 PMC9276481

[9] Ramakrishnan R Rao S He JR. Perinatal health predictors using artificial intelligence: A review. Womens Health (Lond). (2021) 17:17455065211046132. doi: 10.1177/17455065211046132 34519596 PMC8445524

[10] Barua PD Vicnesh J Lih OS Palmer EE Yamakawa T Kobayashi M. Artificial intelligence assisted tools for the detection of anxiety and depression leading to suicidal ideation in adolescents: a review. Cognit Neurodyn. (2022) 18(1):1–22. doi: 10.1007/s11571-022-09904-0 PMC9684805 36467993

[11] Rekik A Nasri A Mrabet S Gharbi A Souissi A Gargouri A. Non-motor features of essential tremor with midline distribution. Neurol Sci. (2022) 43:5917–25. doi: 10.1007/s10072-022-06262-x 35849198

[12] Yuan Y Ran L Lei L Zhu H Zhu X Chen H. The expanding phenotypic spectrums associated with ATP1A3 mutation in a family with rapid-onset dystonia parkinsonism. Neurodegener Dis. (2020) 20:84–9. doi: 10.1159/000511733 33326973

[13] Scorr LM Factor SA Parra SP Kaye R Paniello RC Norris SA. Oromandibular dystonia: A clinical examination of 2,020 cases. Front Neurol. (2021) 12:700714. doi: 10.3389/fneur.2021.700714 34603182 PMC8481678

[14] Przedborski M Sharon D Chan S Kohandel M. A mean-field approach for modeling the propagation of perturbations in biochemical reaction networks. Eur J Pharm Sci. (2021) 165:105919. doi: 10.1016/j.ejps.2021.105919 34175448

[15] Alvarez I Hurley SA Parker AJ Bridge H. Human primary visual cortex shows larger population receptive fields for binocular disparity-defined stimuli. Brain Struct Funct. (2021) 226:2819–38. doi: 10.1007/s00429-021-02351-3 PMC8541985 34347164

[16] Wang J Wang C. The coming Omicron waves and factors affecting its spread after China reopening borders. BMC Med Inform Decis Mak. (2023) 23:186. doi: 10.1186/s12911-023-02219-y 37715187 PMC10503199

[17] Bains A Abraham E Hsieh A Rubin BR Levi JR Cohen MB. Characteristics and frequency of children with severe obstructive sleep apnea undergoing elective polysomnography. Otolaryngol Head Neck Surg. (2020) 163:1055–60. doi: 10.1177/0194599820931084 32539583

[18] Zhang W Yan Y Wu Y Yang H Zhu P Yan F. Medicinal herbs for the treatment of anxiety: A systematic review and network meta-analysis. Pharmacol Res. (2022) 179:106204. doi: 10.1016/j.phrs.2022.106204 35378276

[19] Miklowitz DJ Schneck CD Walshaw PD Singh MK Sullivan AE Suddath RL. Effects of Family-Focused Therapy vs Enhanced Usual Care for Symptomatic Youths at High Risk for Bipolar Disorder: A Randomized Clinical Trial. JAMA Psychiatry. (2020) 77:455–63. doi: 10.1001/jamapsychiatry.2019.4520 PMC6990706 31940011

[20] Vita G Compri B Matcham F Barbui C Ostuzzi G. Antidepressants for the treatment of depression in people with cancer. Cochrane Database Syst Rev. (2023) 3:CD011006. doi: 10.1002/14651858.CD011006.pub4 36999619 PMC10065046

[21] Konvalin F Grosse-Wentrup F Nenov-Matt T Fischer K Barton BB Goerigk S. Borderline personality features in patients with persistent depressive disorder and their effect on CBASP outcome. Front Psychiatry. (2021) 12:608271. doi: 10.3389/fpsyt.2021.608271 33790813 PMC8006327

[22] Takahashi R Takahashi T Okada Y Kohzuki M Ebihara S. Factors associated with quality of life in patients receiving lung transplantation: a cross-sectional study. BMC Pulm Med. (2023) 23:225. doi: 10.1186/s12890-023-02526-0 37353819 PMC10288771

[23] Cooper R Shkolnikov VM Kudryavtsev AV Malyutina S Ryabikov A Arnesdatter Hopstock L. Between-study differences in grip strength: a comparison of Norwegian and Russian adults aged 40-69 years. J Cachexia Sarcopenia Muscle. (2021) 12:2091–100. doi: 10.1002/jcsm.12816 PMC8718040 34605224

[24] Wang X Xiong J Yang J Yuan T Jiang Y Zhou X. Meta-analysis of the clinical effectiveness of combined acupuncture and Western Medicine to treat post-stroke depression. J Tradit Chin Med. (2021) 41:6–16. doi: 10.19852/j.cnki.jtcm.2021.01.002 33522192

[25] Li H Schlaeger JM Patil CL Danciu OC Xia Y Sun J. Feasibility of acupuncture and exploration of metabolomic alterations for psychoneurological symptoms among breast cancer survivors. Biol Res Nurs. (2023) 25:326–35. doi: 10.1177/10998004221136567 PMC10236441 36306737

[26] Bernabei M Bontadi J Sisto L. Stradivari harp tree-ring data. Data Brief. (2022) 43:108453. doi: 10.1016/j.dib.2022.108453 35864874 PMC9294041

[27] Cabezos-Bernal PM Rodriguez-Navarro P Gil-Piqueras T. Documenting paintings with gigapixel photography. J Imag. (2021) 7:156. doi: 10.3390/jimaging7080156 PMC8404936 34460792

[28] Datta G Kundu S Yin Z Lakkireddy RT Mathai J Jacob AP. A processing-in-pixel-in-memory paradigm for resource-constrained TinyML applications. Sci Rep. (2022) 12:14396. doi: 10.1038/s41598-022-17934-1 35999235 PMC9399136

[29] Vanlancker E Vanhoecke B Sieprath T Bourgeois J Beterams A De Moerloose B. Oral microbiota reduce wound healing capacity of epithelial monolayers, irrespective of the presence of 5-fluorouracil. Exp Biol Med (Maywood). (2018) 243:350–60. doi: 10.1177/1535370217753842 PMC6022929 29338309

[30] Liu J Malekzadeh M Mirian N Song TA Liu C Dutta J. Artificial intelligence-based image enhancement in PET imaging: noise reduction and resolution enhancement. PET Clin. (2021) 16:553–76. doi: 10.1016/j.cpet.2021.06.005 PMC8457531 34537130

[31] Niederleithner M de Sisternes L Stino H Sedova A Schlegl T Bagherinia H. Ultra-widefield OCT angiography. IEEE Trans Med Imag. (2023) 42:1009–20. doi: 10.1109/TMI.2022.3222638 36383595

[32] Ma J Wang G Zhang L Zhang Q. Restoration and enhancement on low exposure raw images by joint demosaicing and denoising. Neural Netw. (2023) 162:557–70. doi: 10.1016/j.neunet.2023.03.018 36996687

[33] Feng L Huang ZH Zhong YM Xiao W Wen CB Song HB. Research and application of tongue and face diagnosis based on deep learning. Digit Health. (2022) 8:20552076221124436. doi: 10.1177/20552076221124436 36159155 PMC9490485

[34] Jiang T Hu XJ Yao XH Tu LP Huang JB Ma XX. Tongue image quality assessment based on a deep convolutional neural network. BMC Med Inform Decis Mak. (2021) 21:147. doi: 10.1186/s12911-021-01508-8 33952228 PMC8097848

[35] Su HY Hsieh ST Tsai KZ Wang YL Wang CY Hsu SY. Fusion extracted features from deep learning for identification of multiple positioning errors in dental panoramic imaging. J Xray Sci Technol. (2023) 31:1315–32. doi: 10.3233/XST-230171 37840464

[36] Yang G Zhou S He H Shen Z Liu Y Hu J. Exploring the “gene-protein-metabolite” network of coronary heart disease with phlegm and blood stasis syndrome by integrated multi-omics strategy. Front Pharmacol. (2022) 13:1022627. doi: 10.3389/fphar.2022.1022627 36523490 PMC9744761

[37] Sculco PK Wright T Malahias MA Gu A Bostrom M Haddad F. The diagnosis and treatment of acetabular bone loss in revision hip arthroplasty: an international consensus symposium. HSS J. (2022) 18:8–41. doi: 10.1177/15563316211034850 35082557 PMC8753540

[38] Wu HT Ji CH Dai RC Hei PJ Liang J Wu XQ. Traditional Chinese medicine treatment for COVID-19: An overview of systematic reviews and meta-analyses. J Integr Med. (2022) 20:416–26. doi: 10.1016/j.joim.2022.06.006 PMC9225921 35811240

[39] Yang C Li S Huang T Lin H Jiang Z He Y. Effectiveness and safety of vonoprazan-based regimen for Helicobacter pylori eradication: A meta-analysis of randomized clinical trials. J Clin Pharm Ther. (2022) 47:897–904. doi: 10.1111/jcpt.13637 35247003

[40] Zheng X Qian M Ye X Zhang M Zhan C Li H. Implications for long COVID: A systematic review and meta-aggregation of experience of patients diagnosed with COVID-19. J Clin Nurs. (2024) 33:40–57. doi: 10.1111/jocn.16537 36253950 PMC9874539

[41] Stacchiotti S Miah AB Frezza AM Messiou C Morosi C Caraceni A. Epithelioid hemangioendothelioma, an ultra-rare cancer: a consensus paper from the community of experts. ESMO Open. (2021) 6:100170. doi: 10.1016/j.esmoop.2021.100170 34090171 PMC8182432

[42] Bajwah S Oluyase AO Yi D Gao W Evans CJ Grande G. The effectiveness and cost-effectiveness of hospital-based specialist palliative care for adults with advanced illness and their caregivers. Cochrane Database Syst Rev. (2020) 9:CD012780. doi: 10.1002/14651858.CD012780.pub2 32996586 PMC8428758

[43] Heo MS Kim JE Hwang JJ Han SS Kim JS Yi WJ. Artificial intelligence in oral and maxillofacial radiology: what is currently possible? Dentomaxillofac Radiol. (2021) 50:20200375. doi: 10.1259/dmfr.20200375 33197209 PMC7923066

[44] Dai X Lei Y Wang T Axente M Xu D Patel P. Self-supervised learning for accelerated 3D high-resolution ultrasound imaging. Med Phys. (2021) 48:3916–26. doi: 10.1002/mp.14946 PMC11699523 33993508

[45] Zhuang Q Gan S Zhang L. Human-computer interaction based health diagnostics using ResNet34 for tongue image classification. Comput Methods Prog Biomed. (2022) 226:107096. doi: 10.1016/j.cmpb.2022.107096 36191350

[46] Qiu G Shen Y Cheng K Liu L Zeng S. Mobility-aware privacy-preserving mobile crowdsourcing. Sensors (Basel). (2021) 21:2474. doi: 10.3390/s21072474 33918353 PMC8038310

[47] Hu G Grover CE Arick MA Liu M Peterson DG Wendel JF. Homoeologous gene expression and co-expression network analyses and evolutionary inference in allopolyploids. Brief Bioinform. (2021) 22:1819–35. doi: 10.1093/bib/bbaa035 PMC7986634 32219306

[48] Paterno GB Silveira CL Kollmann J Westoby M Fonseca CR. The maleness of larger angiosperm flowers. Proc Natl Acad Sci U S A. (2020) 117:10921–6. doi: 10.1073/pnas.1910631117 PMC7245066 32366661

[49] Jangam E Annavarapu CSR. A stacked ensemble for the detection of COVID-19 with high recall and accuracy. Comput Biol Med. (2021) 135:104608. doi: 10.1016/j.compbiomed.2021.104608 34247135 PMC8241584

[50] Wu J Zhu D Fang L Deng Y Zhong Z. Efficient layer compression without pruning. IEEE Trans Image Process. (2023) 32:4689–700. doi: 10.1109/TIP.2023.3302519 37561618

[51] Ren Y Sarkar A Veltri P Ay A Dobra A Kahveci T. Pattern discovery in multilayer networks. IEEE/ACM Trans Comput Biol Bioinform. (2022) 19:741–52. doi: 10.1109/TCBB.2021.3105001 34398763

[52] Badias A Banerjee AG. Neural network layer algebra: A framework to measure capacity and compression in deep learning. IEEE Trans Neural Netw Learn Syst. (2023). doi: 10.1109/TNNLS.2023.3241100 37027551

[53] Jiang C Jiang C Chen D Hu F. Densely connected neural networks for nonlinear regression. Entropy (Basel). (2022) 24:876. doi: 10.3390/e24070876 35885098 PMC9317522

[54] Ray I Raipuria G Singhal N. Rethinking imageNet pre-training for computational histopathology. Annu Int Conf IEEE Eng Med Biol Soc. (2022) 2022:3059–62. doi: 10.1109/EMBC48229.2022.9871687 36086630

[55] Xin X Gong H Hu R Ding X Pang S Che Y. Intelligent large-scale flue-cured tobacco grading based on deep densely convolutional network. Sci Rep. (2023) 13:11119. doi: 10.1038/s41598-023-38334-z 37429961 PMC10333347

[56] Zhang M Wen G Zhong J Chen D Wang C Huang X. MLP-like model with convolution complex transformation for auxiliary diagnosis through medical images. IEEE J BioMed Health Inform. (2023) 27:4385–96. doi: 10.1109/JBHI.2023.3292312 37467088

[57] Zhu J Zhang C Zhang C. Papaver somniferum and Papaver rhoeas Classification Based on Visible Capsule Images Using a Modified MobileNetV3-Small Network with Transfer Learning. Entropy (Basel). (2023) 25:447. doi: 10.3390/e25030447 36981335 PMC10047573

[58] Luo Y Zeng Z Lu H Lv E. Posture detection of individual pigs based on lightweight convolution neural networks and efficient channel-wise attention. Sensors (Basel). (2021) 21:8369. doi: 10.3390/s21248369 34960477 PMC8705977

[59] Banerjee A Mutlu OC Kline A Surabhi S Washington P Wall DP. Training and profiling a pediatric facial expression classifier for children on mobile devices: machine learning study. JMIR Form Res. (2023) 7:e39917. doi: 10.2196/39917 35962462 PMC10131663

[60] Hamed Mozaffari M Lee WS. Encoder-decoder CNN models for automatic tracking of tongue contours in real-time ultrasound data. Methods. (2020) 179:26–36. doi: 10.1016/j.ymeth.2020.05.011 32450205

[61] Lin SL. Application combining VMD and resNet101 in intelligent diagnosis of motor faults. Sensors (Basel). (2021) 21:6065. doi: 10.3390/s21186065 34577272 PMC8473405

[62] Wang B Zhang W. ACRnet: Adaptive Cross-transfer Residual neural network for chest X-ray images discrimination of the cardiothoracic diseases. Math Biosci Eng. (2022) 19:6841–59. doi: 10.3934/mbe.2022322 35730285

[63] Choi K Ryu H Kim J. Deep residual networks for user authentication via hand-object manipulations. Sensors (Basel). (2021) 21:2981. doi: 10.3390/s21092981 33922833 PMC8122988

[64] Sumit SS Rambli DRA Mirjalili S Miah MSU Ejaz MM. ReSTiNet: an efficient deep learning approach to improve human detection accuracy. MethodsX. (2022) 10:101936. doi: 10.1016/j.mex.2022.101936 36578294 PMC9791404

[65] Chantrapornchai C Kajkamhaeng S Romphet P. Micro-architecture design exploration template for AutoML case study on SqueezeSEMAuto. Sci Rep. (2023) 13:10642. doi: 10.1038/s41598-023-37682-0 37391458 PMC10313661

[66] Reis HC Turk V Khoshelham K Kaya S. MediNet: transfer learning approach with MediNet medical visual database. Multimed Tools Appl. (2023), 1–44. doi: 10.1007/s11042-023-14831-1 PMC10025796 37362724

[67] Liu T Zhang H Long H Shi J Yao Y. Convolution neural network with batch normalization and inception-residual modules for Android malware classification. Sci Rep. (2022) 12:13996. doi: 10.1038/s41598-022-18402-6 35978023 PMC9385674

[68] Zhu Z Qin J Chen Z Chen Y Chen H Wang X. Sulfammox forwarding thiosulfate-driven denitrification and anammox process for nitrogen removal. Environ Res. (2022) 214:113904. doi: 10.1016/j.envres.2022.113904 35863443

[69] Han T Nebelung S Pedersoli F Zimmermann M Schulze-Hagen M Ho M. Advancing diagnostic performance and clinical usability of neural networks via adversarial training and dual batch normalization. Nat Commun. (2021) 12:4315. doi: 10.1038/s41467-021-24464-3 34262044 PMC8280105

[70] Gong J Liu J Hao W Nie S Wang S Peng W. Computer-aided diagnosis of ground-glass opacity pulmonary nodules using radiomic features analysis. Phys Med Biol. (2019) 64:135015. doi: 10.1088/1361-6560/ab2757 31167172

[71] Zheng C Wang W Young SD. Identifying HIV-related digital social influencers using an iterative deep learning approach. AIDS. (2021) 35:S85–9. doi: 10.1097/QAD.0000000000002841 PMC8059038 33867491

[72] Li D Hu J Zhang L Li L Yin Q Shi J. Deep learning and machine intelligence: New computational modeling techniques for discovery of the combination rules and pharmacodynamic characteristics of Traditional Chinese Medicine. Eur J Pharmacol. (2022) 933:175260. doi: 10.1016/j.ejphar.2022.175260 36116517

[73] Zhang S Zhang M Ma S Wang Q Qu Y Sun Z. Research progress of deep learning in the diagnosis and prevention of stroke. BioMed Res Int. (2021) 2021:5213550. doi: 10.1155/2021/5213550 34414235 PMC8370809

[74] Gul H Mansor Nizami S Khan MA. Estimation of body stature using the percutaneous length of ulna of an individual. Cureus. (2020) 12:e659. doi: 10.7759/cureus.6599 PMC7003724 32064181

[75] Coras R Sturchio GA Bru MB Fernandez AS Farietta S Badia SC. Analysis of the correlation between disease activity score 28 and its ultrasonographic equivalent in rheumatoid arthritis patients. Eur J Rheumatol. (2020) 7:118–23. doi: 10.5152/eurjrheumatol. PMC7431356 32716834

[76] Getu MA Wang P Kantelhardt EJ Seife E Chen C Addissie A. Translation and validation of the EORTC QLQ-BR45 among Ethiopian breast cancer patients. Sci Rep. (2022) 12:605. doi: 10.1038/s41598-021-02511-9 35906247 PMC9338022

[77] Serçe S Ovayolu Ö Bayram N Ovayolu N Kul S. The effect of breathing exercise on daytime sleepiness and fatigue among patients with obstructive sleep apnea syndrome. J Breath Res. (2022) 16:ac894d. doi: 10.1088/1752-7163/ac894d 36004722

[78] Giusca S Wolf D Hofmann N Hagstotz S Forschner M Schueler M. Splenic switch-off for determining the optimal dosage for adenosine stress cardiac MR in terms of stress effectiveness and patient safety. J Magn Reson Imag. (2020) 52:1732–42. doi: 10.1002/jmri.27248 32557923

[79] Hallinan JTPD Zhu L Yang K Makmur A Algazwi DAR Thian YL. Deep learning model for automated detection and classification of central canal, lateral recess, and neural foraminal stenosis at lumbar spine MRI. Radiology. (2021) 300:130–8. doi: 10.1148/radiol.2021204289 33973835

[80] Howard FM Kochanny S Koshy M Spiotto M Pearson AT. Machine learning-guided adjuvant treatment of head and neck cancer. JAMA Netw Open. (2020) 3:e2025881. doi: 10.1001/jamanetworkopen.2020.25881 33211108 PMC7677764

[81] Gichoya JW Banerjee I Bhimireddy AR Burns JL Celi LA Chen LC. AI recognition of patient race in medical imaging: a modelling study. Lancet Digit Health. (2022) 4:e406–14. doi: 10.1016/S2589-7500(22)00063-2 PMC9650160 35568690

[82] Nahm FS. Receiver operating characteristic curve: overview and practical use for clinicians. Korean J Anesthesiol. (2022) 75:25–36. doi: 10.4097/kja.21209 35124947 PMC8831439

[83] Janssens ACJW Martens FK. Reflection on modern methods: Revisiting the area under the ROC Curve. Int J Epidemiol. (2020) 49:1397–403. doi: 10.1093/ije/dyz274 31967640

[84] Jiang YW Xu XJ Wang R Chen CM. Radiomics analysis based on lumbar spine CT to detect osteoporosis. Eur Radiol. (2022) 32:8019–26. doi: 10.1007/s00330-022-08805-4 PMC9059457 35499565

[85] Lalousis PA Wood SJ Schmaal L Chisholm K Griffiths SL Reniers RLEP. Heterogeneity and classification of recent onset psychosis and depression: A multimodal machine learning approach. Schizophr Bull. (2021) 47:1130–40. doi: 10.1093/schbul/sbaa185 PMC8266654 33543752

[86] Labandeira CM Alonso Losada MG Yáñez Baña R Cimas Hernando MI Cabo López I Paz González JM. Effectiveness of safinamide over mood in Parkinson’s disease patients: secondary analysis of the open-label study SAFINONMOTOR. Adv Ther. (2021) 38:5398–411. doi: 10.1007/s12325-021-01873-w PMC8440147 34523075

[87] Kucheryavskiy S Zhilin S Rodionova O Pomerantsev A. Procrustes cross-validation-A bridge between cross-validation and independent validation sets. Anal Chem. (2020) 92:11842–50. doi: 10.1021/acs.analchem.0c02175 32786450

[88] Tsadok I Scheinowitz M Shpitzer SA Ketko I Epstein Y Yanovich R. Assessing rectal temperature with a novel non-invasive sensor. J Therm Biol. (2021) 95:102788. doi: 10.1016/j.jtherbio.2020.102788 33454029

[89] Zhao C Hu S He T Yuan L Yang X Wang J. Sichuan da xue xue bao yi xue ban. Sichuan Da Xue Xue Bao Yi Xue Ban. (2023) 54(5):923–9. doi: 10.12182/20230960303 PMC10579068 37866947

[90] Yang NN Lin LL Li YJ Li HP Cao Y Tan CX. Potential mechanisms and clinical effectiveness of acupuncture in depression. Curr Neuropharmacol. (2022) 20:738–50. doi: 10.2174/1570159X19666210609162809 PMC9878952 35168522

[91] Du Y Zhang L Liu W Rao C Li B Nan X. Effect of acupuncture treatment on post-stroke cognitive impairment: A randomized controlled trial. Med (Baltimore). (2020) 99:e23803. doi: 10.1097/MD.0000000000023803 PMC7748352 33371155

[92] Li YX Xiao XL Zhong DL Luo LJ Yang H Zhou J. Effectiveness and safety of acupuncture for migraine: an overview of systematic reviews. Pain Res Manage. (2020) 2020:3825617. doi: 10.1155/2020/3825617 PMC7125485 32269669

[93] Heidecker B Dagan N Balicer R Eriksson U Rosano G Coats A. Myocarditis following COVID-19 vaccine: incidence, presentation, diagnosis, pathophysiology, therapy, and outcomes put into perspective. A clinical consensus document supported by the Heart Failure Association of the European Society of Cardiology (ESC) and the ESC Working Group on Myocardial and Pericardial Diseases. Eur J Heart Fail. (2022) 24:2000–18. doi: 10.1002/ejhf.2669 PMC9538893 36065751

[94] Leung AK Lam JM Leong KF Hon KL. Tinea corporis: an updated review. Drugs Context. (2020) 9:2020-5-6. doi: 10.7573/17404398 PMC7375854 32742295

[95] Rai B Kaur J Jacobs R Anand SC. Adenosine deaminase in saliva as a diagnostic marker of squamous cell carcinoma of tongue. Clin Oral Investig. (2011) 15:347–9. doi: 10.1007/s00784-010-0404-z 20379753

[96] Dong CW Zhu HK Zhao JW Jiang YW Yuan HB Chen QS. Sensory quality evaluation for appearance of needle-shaped green tea based on computer vision and nonlinear tools. J Zhejiang Univ Sci B. (2017) 18:544–8. doi: 10.1631/jzus.B1600423 PMC5482049 28585431

[97] Li SR Dugan S Masterson J Hudepohl H Annand C Spencer C. Classification of accurate and misarticulated/αr/for ultrasound biofeedback using tongue part displacement trajectories. Clin Linguist Phon. (2023) 37:196–222. doi: 10.1080/02699206.2022.2039777 35254181 PMC9448831

[98] Bommineni VL Erus G Doshi J Singh A Keenan BT Schwab RJ. Automatic segmentation and quantification of upper airway anatomic risk factors for obstructive sleep apnea on unprocessed magnetic resonance images. Acad Radiol. (2023) 30:421–30. doi: 10.1016/j.acra.2022.04.023 PMC10077883 35606257

[99] Juruena MF. Understanding subthreshold depression. Shanghai Arch Psychiatry. (2012) 24:292–3. doi: 10.3969/j.issn.1002-0829.2012.05.009 PMC4198881 25328356

[100] Sherbourne CD Wells KB Hays RD Rogers W Burnam MA Judd LL. Subthreshold depression and depressive disorder: clinical characteristics of general medical and mental health specialty outpatients. Am J Psychiatry. (1994) 151:1777–84. doi: 10.1176/ajp.151.12.1777 7977885

